# Speed of cooling after cardiac arrest in relation to the intervention effect: a sub-study from the TTM2-trial

**DOI:** 10.1186/s13054-022-04231-6

**Published:** 2022-11-15

**Authors:** Rupert F. G. Simpson, Josef Dankiewicz, Grigoris V. Karamasis, Paolo Pelosi, Matthias Haenggi, Paul J. Young, Janus Christian Jakobsen, Jonathan Bannard-Smith, Pedro D. Wendel-Garcia, Fabio Silvio Taccone, Per Nordberg, Matt P. Wise, Anders M. Grejs, Gisela Lilja, Roy Bjørkholt Olsen, Alain Cariou, Jean Baptiste Lascarrou, Manoj Saxena, Jan Hovdenes, Matthew Thomas, Hans Friberg, John R. Davies, Niklas Nielsen, Thomas R. Keeble

**Affiliations:** 1 Essex Cardiothoracic Centre, MSE Trust, Basildon, Essex UK; 2 MTRC, Anglia Ruskin School of Medicine, Chelmsford, Essex UK; 3 Department of Clinical Sciences Lund, Sections of Cardiology, Lund, Sweden; 4 Department of Surgical Sciences and Integrated Diagnostics, University of Genoa, Genoa, Italy; 5 Anesthesiology and Critical Care, San Martino Policlinico Hospital, IRCCS for Oncology and Neurosciences, Genoa, Italy; 6 Department of Intensive Care Medicine, University Hospital Bern, University of Bern, Bern, Switzerland; 7 Intensive Care Unit, Wellington Hospital, Wellington, New Zealand; 8 Medical Research Institute of New Zealand, Wellington, New Zealand; 9 Australian and New Zealand Intensive Care Research Centre, Monash University, Melbourne, VIC Australia; 10 Department of Critical Care, University of Melbourne, Melbourne, VIC Australia; 11 Copenhagen Trial Unit, Centre for Clinical Intervention Research, Copenhagen, Capital Region of Denmark Denmark; 12 Department of Regional Health Research, The Faculty of Health Sciences, University of Southern Denmark, Copenhagen, Denmark; 13 Department of Adult Critical Care, Manchester University NHS Foundation Trust, Manchester, UK; 14 Division of Infection, Immunity and Respiratory Medicine, The University of Manchester, Manchester, UK; 15 Institute of Intensive Care Medicine, University Hospital of Zurich, Zurich, Switzerland; 16 Department of Intensive Care, Hôpital Universitaire de Bruxelles (HUB), Université Libre de Bruxelles (ULB), Brussels, Belgium; 17 Department of Clinical Science and Education, Center for Resuscitation Science, Karolinska Institute, Solna, Sweden; 18 Function Perioperative Medicine and Intensive Care, Karolinska University Hospital, Stockholm, Sweden; 19 Adult Critical Care, University Hospital of Wales, Cardiff, UK; 20 Department of Intensive Care Medicine, Department of Clinical Medicine, Aarhus University Hospital, Aarhus, Denmark; 21 Neurology, Department of Clinical Sciences Lund, Skane University Hospital, Lund University, Lund, Sweden; 22 Department of Anesthesiology, Sørlandet Hospital, Arendal, Norway; 23 Medical Intensive Care Unit, Cochin University Hospital (APHP), Paris Cité University, Paris, France; 24 Medicine Intensive Reanimation, CHU Nantes, Nantes, France; 25 Critical Care Division, The George Institute for Global Health, University of New South Wales, Sydney, Australia; 26 Department of Intensive Care Medicine, Bansltwon-Lidcombe Hospital, South Western Sydney, Sydney, New South Wales Australia; 27 Division of Emergencies and Critical Care, Department of Anesthesiology, Oslo University Hospital, Rikshospitalet, Oslo, Norway; 28 Intensive Care Unit, University Hospitals Bristol and Weston, Bristol, UK; 29 Department of Clinical Science, Intensive and Perioperative Care, Skane University Hospital, Lund University, Malmo, Sweden; 30 Department of Clinical Sciences Lund, Sections of Anesthesiology and Intensive Care, Lund, Sweden

**Keywords:** Out of hospital cardiac arrest, Temperature management, Hypothermia, Time to target temperature

## Abstract

**Background:**

Targeted temperature management (TTM) is recommended following cardiac arrest; however, time to target temperature varies in clinical practice. We hypothesised the effects of a target temperature of 33 °C when compared to normothermia would differ based on average time to hypothermia and those patients achieving hypothermia fastest would have more favorable outcomes.

**Methods:**

In this post-hoc analysis of the TTM-2 trial, patients after out of hospital cardiac arrest were randomized to targeted hypothermia (33 °C), followed by controlled re-warming, or normothermia with early treatment of fever (body temperature, ≥ 37.8 °C). The average temperature at 4 h (240 min) after return of spontaneous circulation (ROSC) was calculated for participating sites. Primary outcome was death from any cause at 6 months. Secondary outcome was poor functional outcome at 6 months (score of 4–6 on modified Rankin scale).

**Results:**

A total of 1592 participants were evaluated for the primary outcome. We found no evidence of heterogeneity of intervention effect based on the average time to target temperature on mortality (*p* = 0.17). Of patients allocated to hypothermia at the fastest sites, 71 of 145 (49%) had died compared to 68 of 148 (46%) of the normothermia group (relative risk with hypothermia, 1.07; 95% confidence interval 0.84–1.36). Poor functional outcome was reported in 74/144 (51%) patients in the hypothermia group, and 75/147 (51%) patients in the normothermia group (relative risk with hypothermia 1.01 (95% CI 0.80–1.26).

**Conclusions:**

Using a hospital’s average time to hypothermia did not significantly alter the effect of TTM of 33 °C compared to normothermia and early treatment of fever.

**Supplementary Information:**

The online version contains supplementary material available at 10.1186/s13054-022-04231-6.

## Introduction

Out of hospital cardiac arrest (OHCA) carries a poor prognosis and for patients admitted to intensive care less than half survive to hospital discharge [1]. Animal models of cardiac arrest suggest that therapeutic hypothermia (TH) is neuro-protective and decreases neuronal injury [2, 3]. Targeted temperature management (TTM 33–36 °C) has become incorporated into major international guidelines [4, 5] in the management of the post OHCA patient. Early work suggesting better neurological recovery and mortality benefit in TTM 33 °C post OHCA from ventricular arrythmia [6, 7] has not been replicated in the investigation of the comparison of TTM 33 °C to TTM 36 °C or duration of cooling (24 or 48 h) [8, 9]. The most recent trial, TTM2, showed that TTM 33 °C when compared to normothermia had no survival or functional outcome benefit at 6 months follow-up [1].

Speed of achieving target temperature remains an area of interest and undetermined therapeutic effect. In vivo data suggest benefits from rapid early cooling, with a 15 min delay in therapeutic hypothermia (TH) post cardiac arrest in canine models associated with worse functional outcomes [10]. To date, no dedicated trials have addressed the question, and the scientific literature based on registry data is variable. Previous work on speed of cooling has suggested that for each 30 min delay to cooling the odds of a poor neurological outcome increases [11]. Contrasting to this, faster reductions in body temperature [12] and a quicker time period to TH [13] have been shown to be associated with worse neurological outcomes. The inherent challenges in the chain of resuscitation from OHCA means there are inevitable delays to cooling. Trials showing benefits to TTM33^o^C were not specifically designed to assess the speed of cooling to target temperature and had median times to target temperature ranging from 120 min to 8 h [6, 7, 14].

Accordingly, we conducted a post-hoc analysis of the TTM-2 trial to investigate if time to target temperature influenced the intervention effect. We hypothesized that those patients cooled to a temperature of 33 °C fastest might have a higher incidence of survival and improved outcomes when compared to a normothermia cohort.

## Methods

The design of the TTM2 trial has been previously published [15]; this was a multi-center, international, randomized, parallel group superiority trial. Inclusion criteria were adults over the age of 18 years admitted with OHCA of presumed cardiac cause who had been admitted to hospital, irrespective of initial rhythm and who were unconscious and not able to verbalize. After eligibility screening, patients were randomized in a 1:1 ratio to undergo hypothermia (TTM33^o^C) or normothermia. Written informed consent was waived, deferred, or obtained from a legal surrogate and was obtained from each patient who regained mental capacity. The study protocol was approved by the ethics committee in each participating country and in line with the declaration of Helsinki.

In this post-hoc study, we focused on speed at which participating sites cooled patients randomized to hypothermia. We chose to only include sites that randomized at least 10 patients to hypothermia to obtain a reasonable estimate of their system-wide (both pre-hospital and in-hospital) capacity of rapidly lowering body temperature after cardiac arrest. We assessed speed of cooling by calculating each site’s average temperature at 4 h post ROSC in the TTM-2-trial. A specific time point, rather than the hourly decrease in temperature was used as initial recordings of temperature (at hour 0) might be affected by delays to core temperature measurement rather than cooling speeds. This time point also harmonizes with a pre-defined post-ischemic cooling period in upcoming clinical trials in a similar area [16].

### Ranking

In this sub-group analysis, we assessed the mean temperature of all patients site by site within 4 h of ROSC. Sites were ranked according to the mean temperature at 4 h among patients randomized to hypothermia and divided into 6 groups. Group 1 had a mean temperature of < 34 °C at 4 h, defining the sites that cooled patients most rapidly and mean temperature increased with group number (groups 2 to 6). The number of groups was chosen based on the number of participants in the first group resulting in a total of six.

The temperature target was consistent with standard practice pre TTM-2 at participating sites and as per international guidelines [4, 17]. The timing interval differs from the original report of the trial results, where the 40-h period of intervention began at the time of randomization, rather than from ROSC. Patients assigned to undergo hypothermia were cooled with a surface or intravascular temperature management device to a target temperature of below 34 degrees. The time from ROSC to randomization was rounded to the nearest hour. The participants from sites with the most rapid cooling in group 1 came from six sites in Sweden, Norway, Switzerland and the United Kingdom.

### Outcomes

The primary outcome was death from any cause at 6 months. The main secondary outcome was poor functional outcome at 6 months, defined as a score of 4–6 on the modified Rankin scale by a structured assessment) [18, 19] along with a binary assessment based on all available data including medical records [1].

### Statistical analysis

We investigated subgroup effects using a generalized linear model with a log-link to estimate the relative risk of death in the hypothermia group adding hospital rank group (groups 1–6) and an interaction between these variables. We assumed that the interaction effect would be linear. Although we recognize that the analysis may be underpowered, the conclusions of this study are based on the interaction. Subgroup results are further described using risk ratios for death and 95% confidence intervals. Participants randomized to hypothermia were compared to participants randomized to normothermia at the selected sites. All analyses are based on generic site effect (i.e., a baseline variable) and not indicative of an individual’s cooling time. All sites time to target temperature is influenced by their case mix of patients and geography; this is an accepted confounder in our analysis.

Standardized risk ratios (RR) were estimated using logistic regression and 95% confidence intervals were calculated using bootstrap resampling, including the design variables (age, initial rhythm, shock on admission, sex, and time to ROSC) as covariates. For survival analysis Cox regression with 95% confidence intervals was used and survival curves are Kaplan–Meier estimates. The proportional hazard assumptions were assessed through inspection of Schoenfeld residuals. Finally, when describing survival data of the hypothermia group of patients we divided them into 4 groups, based on de-facto speed of cooling quartiles, rather than site specific cooling.

## Results

A total of 1592 patients from 35 sites were included in the primary outcome analysis (86% of the full TTM2 trial). Patients recruited from sites where the mean temperature in hypothermia-assigned patients at 4 h post ROSC was < 34 degrees were designated as belonging to ‘group 1’ (the most rapidly cooled group of patients) (Table 1). The remaining patients were split into similar sized groups (variance due to differing number of randomizations per site) and the mean temperature at 4 h of each group increased from groups 2 through to 6 (Table 2).

**Table 1:** Baseline characteristics of group 1, the most rapidly cooled patients, with an average temperature of < 34 °C at 4 h (240 min) post ROSC

	Normothermia	Hypothermia
Total number	150	146
Age (mean (SD))	63.77 (12.80)	65.08 (12.74)
Sex = male (%)	120 (80.0)	113 (77.4)
Body weight (mean (SD))	83.11 (14.96)	83.82 (17.16)
BMI (mean (SD))	27.07 (5.12)	27.40 (5.38)
Hypertension—no. (%)	52 (47.3)	55 (49.5)
Myocardial infarction—no. (%)	27 (31.8)	30 (35.3)
PCI—no. (%)	30 (34.1)	24 (30.0)
Coronary artery bypass grafting—no. (%)	17 (22.7)	12 (17.6)
Heart failure—no. (%)	22 (27.8)	13 (18.8)
NYHA III or IV heart failure—no. (%)	1 (10.0)	3 (37.5)
Median Charlson comorbidity index (median [IQR])	3.00 [1.00, 4.00]	3.00 [2.00, 4.00]
*Location at cardiac arrest *(%)		
Home	71 (47.3)	88 (60.3)
Other	23 (15.3)	13 ( 8.9)
Public place	56 (37.3)	45 (30.8)
Bystander performed CPR (%)	122 (81.3)	116 (79.5)
Bystander witnessed cardiac arrest	137 (91.3)	131 (89.7)
*Initial rhythm* (%)		
Asystole	15 (10.0)	26 (17.8)
Non-perfusing VT	2 (1.3)	3 ( 2.1)
PEA	17 (11.3)	24 (16.4)
ROSC after bystander defibrillation	5 (3.3)	0 ( 0.0)
Unknown—No shock administered	1 (0.7)	2 ( 1.4)
Unknown—Shock administered	3 (2.0)	5 ( 3.4)
VF	107 (71.3)	86 (58.9)
Median time from cardiac arrest to ROSC (min, median [IQR])	27.50 [15.25, 38.75]	27.00 [18.00, 40.75]
Median time from cardiac arrest to randomization (min, mean (SD))	124.13 (47.51)	127.68 (43.75)
Admission tympanic temperature (mean (SD))	35.26 (1.24)	34.70 (1.32)
Admission FOUR motor score (median [IQR])	0.00 [0.00, 0.00]	0.00 [0.00, 0.00]
Bilateral corneal reflexes present (no. %)	17 (23.0)	15 (21.7)
Bilateral pupillary reflexes present (no. %)	65 (59.6)	71 (67.0)
Admission arterial pH (mean (SD))	7.17 (0.16)	7.16 (0.18)
Admission arterial lactate mmol/l (median [IQR])	5.70 [2.80, 8.55]	5.60 [2.80, 8.20]
Shock on admission = (no. (%)	44 (29.3)	44 (30.1)
ST segment elevation myocardial infarction (no. (%)	68 (45.9)	59 (40.7)

SD, standard deviation; BMI, body mass index; PCI, percutaneous coronary intervention; CPR, cardio-pulmonary resuscitation; VT, ventricular tachycardia; VF, ventricular fibrillation; PEA, pulseless electrical activity; ROSC, return of spontaneous circulation

**Table 2:** Results of analysis of patients grouped by average temperature at 4 h

	*n*	Risk ratio	Lower 95% CI	Upper 95% CI	*p* value	Avg temp at 4 h
Group 1—33.8 °C	293	1.07	0.84	1.36	0.64	33.8
Group 2—34.1 °C	270	1.00	0.79	1.27	1.00	34.1
Group 3—34.4 °C	315	1.12	0.89	1.41	0.37	34.4
Group 4—34.8 °C	205	1.10	0.82	1.47	0.58	34.8
Group 5—35.1 °C	257	0.99	0.78	1.26	1.00	35.1
Group 6—35.3 °C	252	0.81	0.64	1.04	0.13	35.3

As group number increases so does average temperature at 4 h

*n*, number of patients per group; CI, confidence interval; Avg, average

### Heterogeneity

The interaction analysis between cooling speed group and the intervention showed a risk ratio of 0.96 (95% CI 0.90–1.02, *p* = 0.17). The direction of the interaction point estimate indicated an increased probability of death in the faster cooling groups (Additional file 1: Fig. 1). When the cooling groups were handled as categorical, rather than a continuous predictor the *p* value for the interaction was 0.52.

**Fig. 1:**
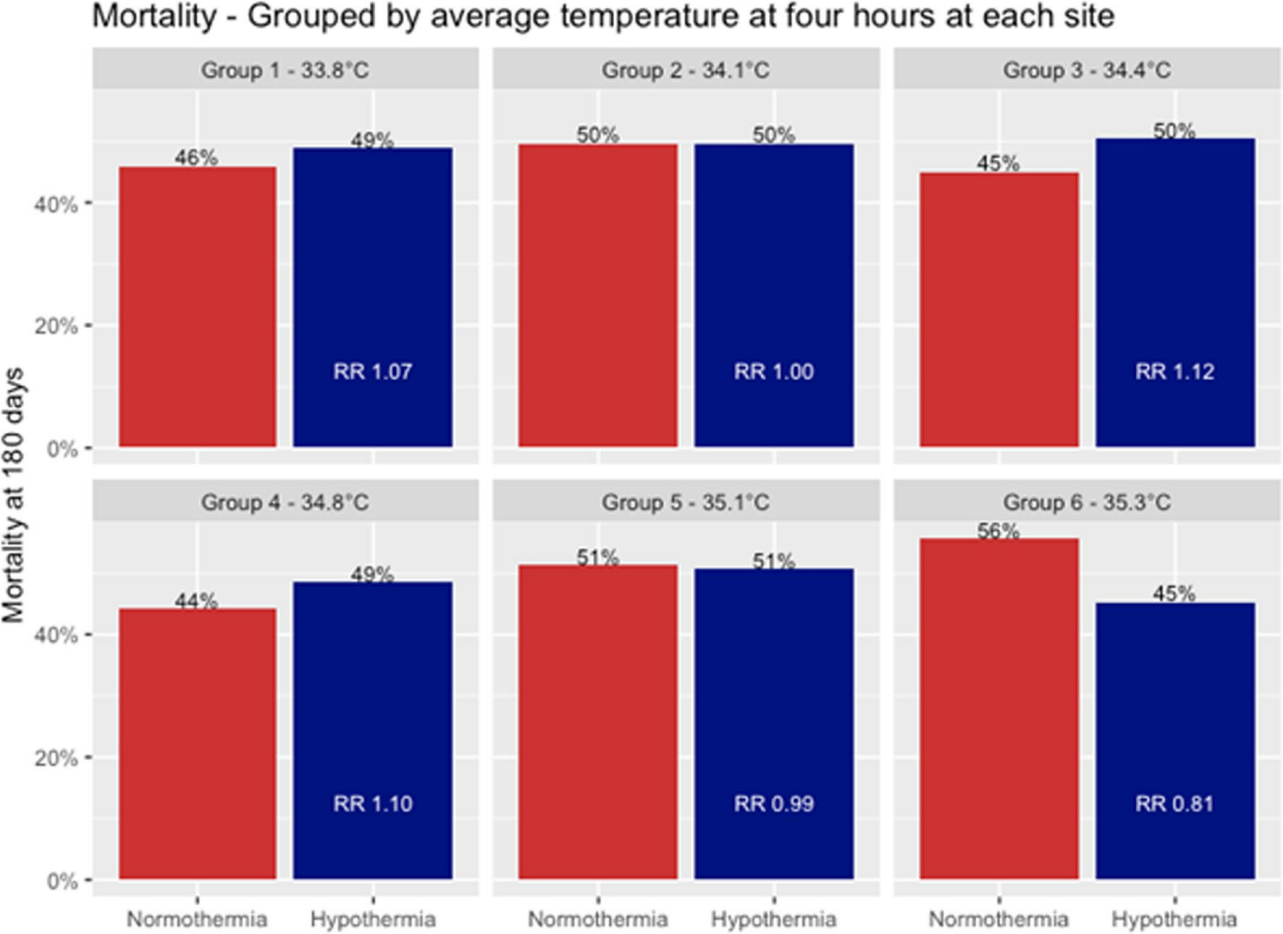
Mortality at 180 days (6 months) across the six groups. Red denotes normothermia patients, and blue denotes hypothermia. There was no trend to suggest that achieving targeted hypothermia faster had a positive effect on mortality outcomes (overall interaction effect, *p* = 0.17). RR = Risk Hypothermia RR, 1.16 (0.93–1.46), Cooling category RR 1.03 (0.99–1.08), Interaction Hypothermia: Cooling Category RR 0.96 (0.90–1.02)

Risk ratios for death for patients randomized to hypothermia varied considerably over the different cooling groups. (Group 1, mean temperature at 4 h 33.8 °C, RR 1.07, 95% CI 0.84–1.36, group 6 mean temperature at 4 h 35.3 °C, RR 0.81, 95% CI 0.64 to 1.04) (Fig. 1).

### Results at fastest sites

**Fig. 2:**
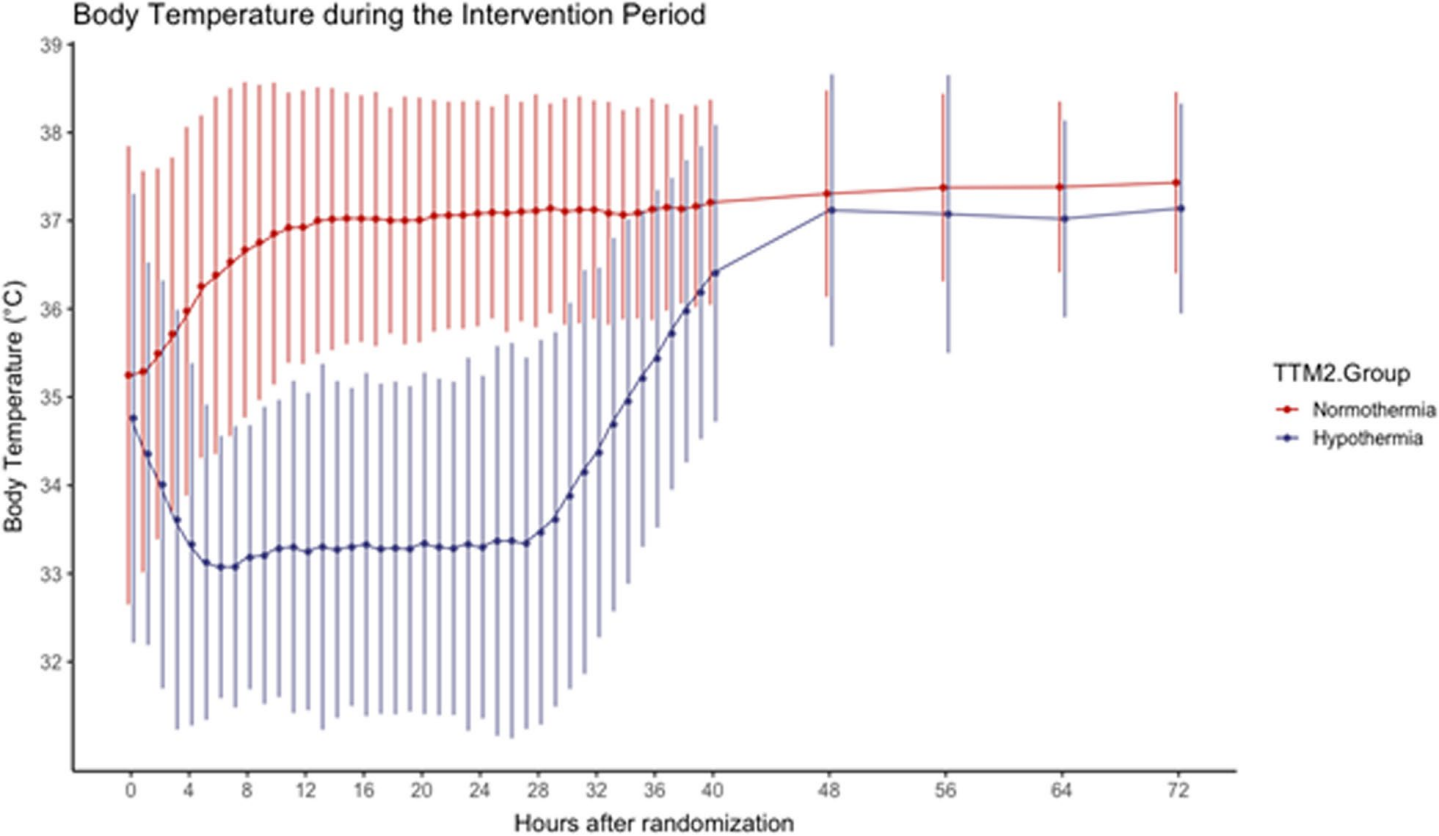
Shown are the body-temperature curve in the hypothermia (blue) and normothermia (red) arms of ‘group 1’ patients, that is those patients with average temperature < 34 at 4 h after ROSC

The six sites with an average temperature ≤ 34 °C at 4 h randomized 296 participants (16% of the trial intention-to-treat population). 150 were assigned to hypothermia and 146 to normothermia, and baseline characteristics for these patients are shown in Table 1, temperature curves for the group are shown in Fig. 2. At 180 days, 68 of 148 (46%) participants in the normothermia group and 71 of 145 (49%) in the hypothermia group had died (RR in the hypothermia group 1.07 (95% CI 0.84–1.36), and survival status was missing for 3 participants. In an adjusted analysis, the risk ratio for death at 180 days was 0.93 (95% CI 0.76–1.14, *p* = 0.50).

Functional outcome was available for 271 (92%) of group 1 participants. In the hypothermia group, 74/144 (51%) had a poor outcome, and in the normothermia group, 75/147 (51%) had a poor outcome (RR with hypothermia 1.01 (95% CI 0.80–1.26). After adjusting for design variables, the risk ratio was 0.89 (95% CI 0.73–1.07, *p* = 0.21).

### Characteristics of patients who were cooled fast

**Fig. 3:**
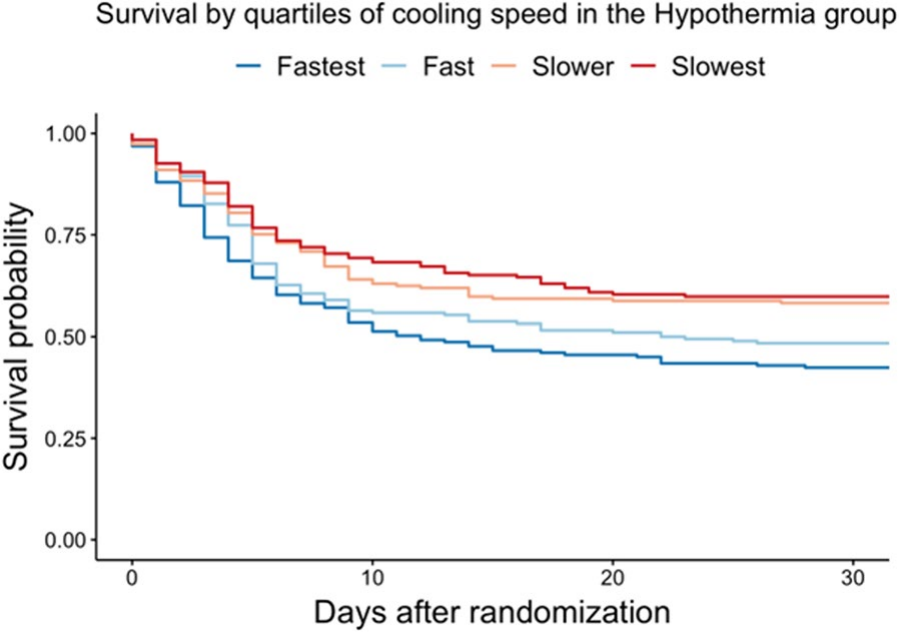
Probability of survival until 30 days after randomization in the hypothermia group. Shown is a Kaplan–Meier curve estimating probability of survival until 30 days in the hypothermia group. The hypothermia group was divided into 4 based on quartiles. Slower cooling speed yielded a lower probability of death at 30 days

When participants in the hypothermia group at all sites were divided by whether they were at or below 34 °C at 4 h, (fast or slow group) participants in the fast group were older (65.6 vs 63.7 years, *p* = 0.043) and had a lower BMI (26.3 vs 28.0, *p* < 0.001). Participants in the fast group had a faster time from cardiac arrest to randomization (125 min vs 142 min, *p* < 0.001) and a lower temperature at admission (34.9 vs. 35.5 °C, *p* < 0.01). In an exploratory non-adjusted analysis, the RR for death in fast group was 1.22, with a 95% CI of 1.07 to 1.39. Using cox regression to analyze survival data a slower cooling speed yielded a lower hazard of death (HR 0.58, 95% CI 0.43–0.77) for the slowest cohort compared to the fastest, *p* < 0.001), Fig. 3.

## Discussion

In this post-hoc analysis of the TTM-2 trial, we calculated the average temperature of hypothermia participants at 4 h at each site and created six groups, based on rapidity of cooling. All hypothermia participants were compared with participants randomized to normothermia in each group. There was no significant difference in outcomes with respect to death or poor functional outcome at 6 months between the hypothermia and normothermia groups in any of the six groups.

It is biologically plausible that faster cooling leads to a neuroprotective effect after resuscitated cardiac arrest with hypoxic brain injury. Randomizing a time-delay in cooling is difficult as individual patients’ temperature trajectories in a randomized trial will vary due to patient characteristics such as severity of illness and body weight, and logistics on any given day. To assess the potential benefit of faster cooling, and simultaneously keeping the randomization from the original trial, we compared outcomes at hospitals with different cooling speeds, considering the entire delay from return of spontaneous circulation. We assessed possible heterogeneity of intervention effects in the TTM2-trial by comparing the trial outcomes in six different groups of hospitals.

We used the hospital rank both as a categorical variable and as a continuous variable and in one analysis found an interaction which could be interpreted as significant with a borderline *p* value. The direction of this interaction favored better outcomes in the hypothermia group with slower cooling. Further investigation showed no clear pattern in the variation of the risk ratio among the different speed of cooling groups. When analyzing only patients that were included at sites which on average cooled patients to < 34 °C at 4 h survival was lower in the hypothermia group.

In this report, we have tried to preserve randomization by performing a subgroup analysis that includes all sites that cooled over 10 patients. The results of this study do not support a mortality benefit or improved functional outcome for patients in the subgroup of patients who were rapidly cooled, as compared to a normothermia group. This aligns with the results of the main TTM2-trial. Within the hypothermia arm of this sub-study, the quartile of patients who achieved 34 °C the fastest had worse mortality outcomes at 30 days.

Results from previous work on time to target hypothermia have been varied; however, there is precedent to support our findings. Haugk et al. conducted a single center retrospective cohort study examining the time to achieve a temperature of < 34 °C from ROSC [12]. The cohort was divided into tertiles of time from ROSC and in each advancing tertile there was an 86% increase in odds of a good neurological outcome, whereas a faster decline in body temperature was associated with a less favorable neurological outcomes. Perman et al. conducted a multi-center retrospective study assessing different time periods in the chain of post arrest temperature management, most notably ‘induction’ (time of onset of therapeutic hypothermia to arrival at target temperature) of TTM [13]. Patients were categorized by length of induction time (< 120 min, 120–300 min and > 300 min) and they found that an induction time > 300 min was associated with better neurological outcomes when compared to those with a faster induction time of < 120 min. Methodology differed to that in our study, as we targeted time from ROSC to target temperature as we felt that this represented the true potential beneficial time that would afford the best possible neuro-protective effect of cooling. Nevertheless, this does show a general trend correlating with our findings of faster cooling speeds do not confer any survival, or functional benefit.

Contrasting to our results, prior work by Sendelbach et al. [11] conducted a secondary data analysis of post arrest time to target temperature and found that the odds of a poor neurological outcome increased for every 30 min delay to target temperature, however this was not statistically significant. Wolff et al. [20] found that time to target temperature and time to coldest temperature were both independent predictors for good neurological outcome. These studies were not randomized and lacked a control cohort of normothermia. The Wolff study in particular had several methodological problems, such as not including the time from cardiac arrest to ROSC as a co-variate in statistical modelling [21].

Pre-hospital cooling and aggressive cooling directly in the cardiac catheter laboratory are effective in decreasing time to target temperature [22–24]; however, a positive effect on neurological outcomes and mortality has not been shown in randomized controlled trials [25, 26]. Furthermore, there are data indicating a signal of harm with infusion of cold IV fluids in pre-hospital cooling [26, 27]. Mounting evidence from large randomized trials [1, 8] does not support the theoretical and neuro-protective benefits seen upon immediate cooling in animal models [28], or the initial favorable outcomes of TH in humans [6, 7] and systematic review has shown these data to be of a low certainty of evidence [29].

Observing the hypothermia group separately, faster cooling was associated with increased mortality at 30 days. The hypothalamus and brainstem are responsible for temperature regulation [30]; it is possible that those patients that achieved 34 °C fastest had a more extensive and devastating hypoxic-ischaemic brain injury with resultant loss of thermoregulatory control and worse outcomes may have been expected regardless of rate of achieving TH. Moreover, in our study, there was a trend towards higher survival rates in those patients with a higher temperature at 4 h.

Our study had several limitations. First, there is the inherent limitation of this being a post hoc analysis without pre-specified grouping and our analyses are presumably underpowered, the original study design of the TTM-2 trial was not devised to specifically answer the question on time to target temperature and outcomes. We have however aimed to maintain an element of randomization by assessing speed of cooling as a site effect in an interaction analysis. Secondly, although rapid cooling to < 34 °C within 4 h of ROSC is achievable in clinical practice, this represents a significant challenge for the majority of sites. In the TTM-2 trial, patients were cooled at a similar rate, or faster than previous trials [6, 7, 9, 14]; however, cooling to below 34 °C within 4 h from ROSC may not be easily achieved or reproducible throughout different healthcare systems. Thirdly, this study does not answer the question of if ultra-early, pre-hospital peri-arrest cooling is of benefit. Previous work in this area has signaled that whilst pre-hospital cooling decreases the time to hypothermia, there is no survival or neurological outcome benefit [25, 26]. Finally, the TTM-2 trial included all patients who had suffered cardiac arrest from a presumed cardiac cause, or unknown cause, irrespective of presenting rhythm therefore the data presented does not specify our findings to a particular mode of arrest.

## Conclusion

In the TTM2-trial population, we did not find an interaction which would indicate better outcomes with hypothermia for patients included at hospitals with overall faster cooling within the first 4 h after ROSC compared to normothermia and early treatment of fever. In an analysis of hypothermia patients only, faster cooling was associated with higher mortality. This association may be due to confounding on the extent of hypoxic-ischaemic brain injury, and subsequent loss of thermoregulatory control.

## Supplementary Information

**Additional file 1: Fig. 1**. Predicted probability of death across the 6 hypothermia groups as compared to the normothermia groups at the corresponding sites. Patients in group 1, with the lowest average temperature at 4 hours had a higher probability of death.

## Data Availability

The study protocol and data-sets analysed for this current study are part of the original TTM-2 trial. They are published [1] and available at NEJM.org.
