# Supplementary figures and images for: Speed of cooling after cardiac arrest in relation to the intervention effect: a sub-study from the TTM2-trial
Source: Crit Care. 2022 Nov 15;26:356. doi: 10.1186/s13054-022-04231-6 (PMC9667681; doi:10.1186/s13054-022-04231-6)

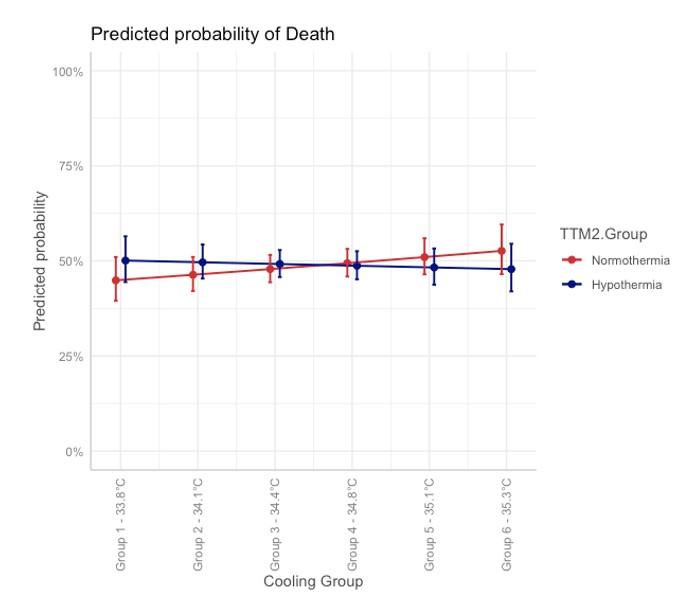

Supplement: Supplementary file 1 — Additional file 1: Fig. 1. Predicted probability of death across the 6 hypothermia groups as compared to the normothermia groups at the corresponding sites. Patients in group 1, with the lowest average temperature at 4 hours had a higher probability of death. [file 13054_2022_4231_MOESM1_ESM.jpg]
